# Efficacy and safety of interferon-alpha 1b injection into the intervertebral foramen with ultrasonic guidance in patients with postherpetic neuralgia: study protocol for a randomized, double-blind, placebo-controlled, multicenter clinical trial

**DOI:** 10.3389/fneur.2024.1516262

**Published:** 2025-01-06

**Authors:** Hui-Min Hu, Wen-Hui Liu, Chen Li, Qing Shi, Chun-Hua Liu, An-Xiang Liu, Yi-Fan Li, Yi Zhang, Peng Mao, Bi-Fa Fan

**Affiliations:** ^1^Graduate School, Beijing University of Chinese Medicine, Beijing, China; ^2^Department of Pain Medicine, China-Japan Friendship Hospital, Beijing, China

**Keywords:** postherpetic neuralgia, interferon-alpha 1b, foraminal injection, randomized controlled trail, protocol

## Abstract

**Purpose:**

Postherpetic neuralgia (PHN) is a type of refractory neuropathic pain that causes significant suffering, disability, economic loss, and medical burden. In this study, we aim to evaluate the efficacy and safety of interferon (IFN)-α1b injection into the intervertebral foramen of patients with PHN.

**Patients and methods:**

This is a study protocol for a randomized, double-blind placebo-controlled multicenter clinical trial. A total of 200 participants with PHN from 11 hospitals will be recruited and randomly assigned to the treatment group administered with IFN-α1b and control group treated with placebo in a 1:1 ratio. Both groups will also receive oral pregabalin 150 mg twice daily and lidocaine injection into the intervertebral foramen as conventional therapy. This trial will involve a screening period, a 2-week intervention, and a 3-month follow-up. The primary outcomes will include the visual analog scale score and duration of pain relief. The secondary outcomes will include the 36-item short-form, dosage and duration of painkillers taken, viral load of varicella-zoster virus DNA, humoral cytokine level, and dosage and frequency of rescue medication. All adverse events and severe adverse events will be assessed during the study.

**Conclusion:**

This study is expected to provide evidence for the efficacy and safety of IFN-α1b injection into the intervertebral foramen in patients with PHN.

**Clinical trial registration:**

https://www.chictr.org.cn/indexEN.html, identifier ChiCTR240008996.

## 1 Introduction

Postherpetic neuralgia (PHN) is the most frequent chronic complication of herpes zoster (HZ), which results from the reactivation of latent varicella-zoster virus (VZV) within the dorsal root ganglia (DRG) (1, 2). PHN is defined as pain lasting >90 days after the onset of an acute HZ rash. In China, approximately one-third of individuals with HZ develop PHN (3). Patients with PHN often report different types of pain and characteristic sensory perturbations (such as tingling or pricking sensation, pain on light touch, pain on cold/heat sensation, electric shock-like pain, numbness, and pain on slight pressure). PHN is a highly burdensome condition for patients, families, and society and has major implications for public health (4).

No disease-modifying therapy is currently available for PHN, and treatment is focused on symptom control. Gabapentin, pregabalin, and tricyclic antidepressants (TCAs) are recommended as first-line oral drugs for treating PHN (5). However, clinical studies have found that gabapentin and pregabalin cause adverse reactions including dizziness, drowsiness, dry mouth, weight gain, and edema (6). The use of TCAs increases the incidence of cardiovascular disease. Topical therapies, such as lidocaine and capsaicin patches, alleviate pain only in patients without skin lesions on painful sites (7, 8). Consequently, there is a dire need for novel therapies that are effective and have good tolerability, to mitigate the impact of PHN on patients’ lives.

The activation and replication of VZV are key mechanisms in the occurrence of PHN. VZV detection rate in patients with PHN can reach 3/4 within 2–6 months after disease onset, and can still be detected up to 2 years later (9). Therefore, we inferred that VZV may directly cause severe and irreversible damage to the nervous system during the pathogenesis of PHN, indirectly promoting the formation of PHN.

Interferons (IFNs), a family of cytokines produced by immune cells after a viral infection, bind to cell surface receptors to produce various antiviral proteins, which act on multiple stages of virus replication to achieve multi-target inhibition of virus replication (10). IFN-α1b is a new type of drug with independent intellectual property rights in China having antiviral, antitumor, and immune regulation functions (11, 12). However, evidence-based multicenter clinical research on the efficacy and safety of IFN-α1b injection into the foramen is still lacking. This study will adopt a multicenter, randomized, double-blind, placebo-controlled experimental design to test the efficacy and safety of IFN-α1b injection into the intervertebral foramen, for treating PHN.

## 2 Methods and analysis

### 2.1 Study design and setting

**Figure 1:**
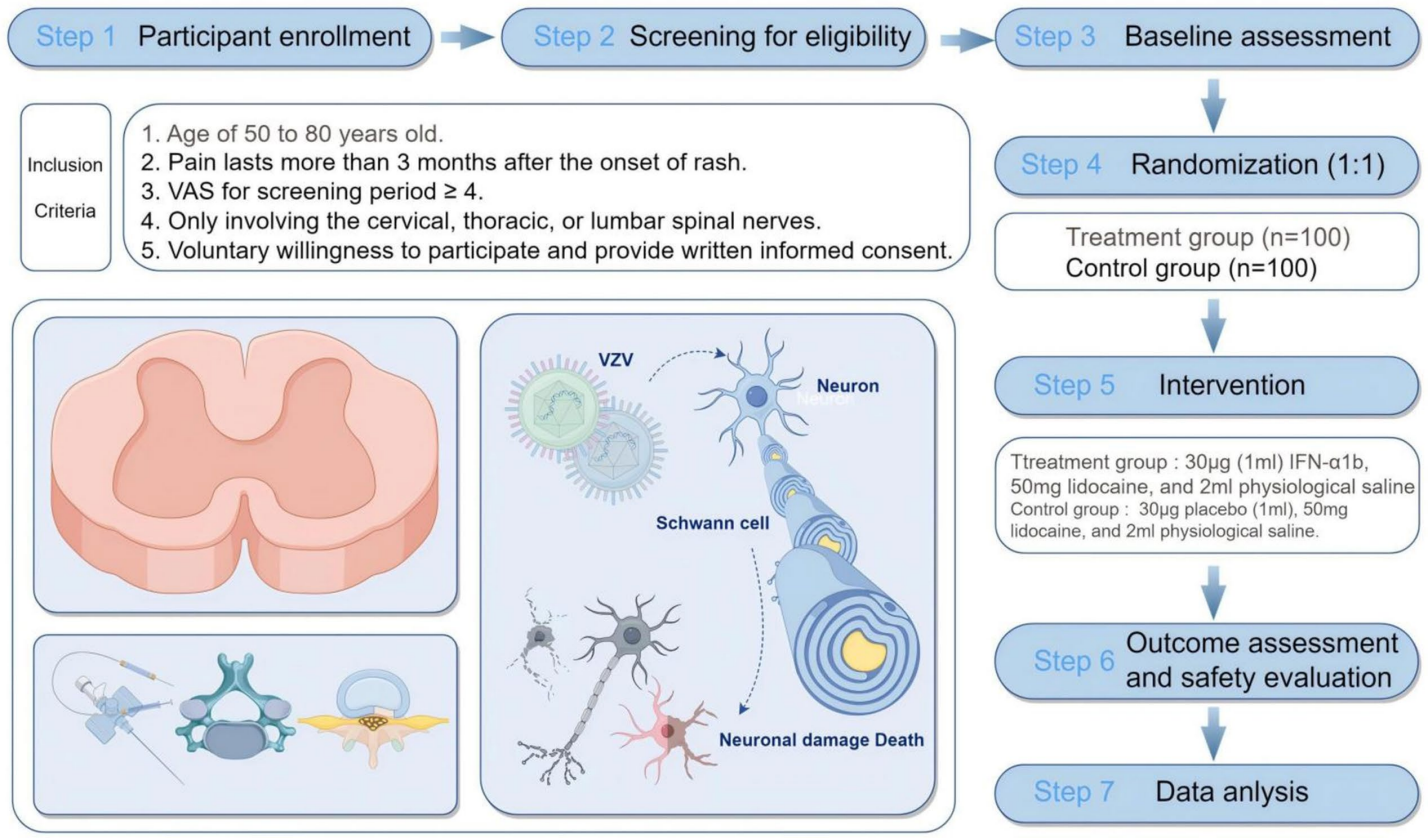
Flowchart of the experimental: efficacy and safety of ultrasound-guided injection of interferon-alpha1b (IFN-alpha1b) into foramen of patients with postherpetic neuralgia.

This study will be a randomized, double-blind, placebo-controlled multicenter clinical trial designed to evaluate the efficacy and safety of IFN-α1b injection into the foramen in patients with PHN. This study will be performed at 11 hospitals in China: China-Japan Friendship Hospital, First Affiliated Hospital of Chongqing Medical University, CR & WISCO General Hospital, Second Affiliated Hospital of Guangzhou Medical University, First Hospital of Lanzhou University, First Affiliated Hospital of Xiamen University, Affiliated Hospital of Xuzhou Medical University, Yanbian University Hospital, Nanjing Drum Tower Hospital, Jilin Provincial People’s Hospital, and Gansu Provincial Hospital. Two-hundred participants who meet the inclusion criteria but not the exclusion criteria will be randomly divided into treatment and control groups in a 1:1 ratio. The study will include a baseline period, a 2-week treatment, and a 3-month follow-up after enrollment. Individual researchers will screen subjects who meet the inclusion criteria. Subjects who do not meet the exclusion criteria will be included in this study. Collection of baseline information will be completed through case report form (CRF) during the baseline period. The experimental flowchart is shown in Figure 1 by Figdraw. The schedules for enrollment, intervention, and assessment are listed in Table 1.

**Table 1:** Schedule of enrolment, treatments, and assessments.

Procedure	Screening period	Treatment period		Follow-up period		
	Visit 1	Visit 2	Visit 3	Visit 4	Visit 5	Visit 6
	Day −3 to 0	Day 7 (After 2 treatments)	Day 14 (After 4 treatments)	Day 30 after treatment	Day 60 after treatment	Day 90 after treatment
Enrolment						
Informed consent	•					
Demographic information	•					
Vital sign	•	•	•	•	•	•
Physical examination	•	•	•	•	•	•
Imaging test	•					
ECG	•					
Laboratory test	•		•			
Randomization	•					
Intervention						
Treatment group		Inject interferon into intervertebral foramen 2 times per week				
Control group		placebo interferon into intervertebral foramen 2 times per week				
Assessment						
VAS	•	•	•	•	•	•
Duration of pain relief	•	•	•	•	•	•
SF-36	•	•	•	•	•	•
Virus load of VZV DNA	•		•			
humoral cytokine level	•		•			
Drug consumption	•	•	•	•	•	•
Adverse events		•	•	•	•	•

ECG, electrocardiogram; VAS, visual analogue scale; SF-36, 36-item short-form; VZV, varicella-zoster virus. Indicate completion or inclusion of the content.

### 2.2 Sample size

A preliminary study with a small sample size and ≥ 25% reduction in VAS after one treatment as the primary outcome showed that the response rate was 46% in the treatment group and 23% in the control group. With *α* = 0.05 (bilateral) and power = 0.90, PASS17 software suggested that at least 85 subjects should be included in each group. Assuming a loss to follow-up rate of 15%, the adjusted sample size should be 100.

### 2.3 Inclusion criteria

Participants who meet all the following requirements will be enrolled:

1. Aged 50–80 years.
2. Pain lasting for >3 months after the onset of rash.
3. VAS score ≥ 4 during the screening period.
4. Only involving the cervical, thoracic, or lumbar spinal nerves.
5. Voluntarily willing to participate and provide written informed consent.

### 2.4 Exclusion criteria

Participants who meet one of the following requirements will be excluded:

1. Head, face, and special area affected by HZ.
2. Puncture site affected by HZ.
3. Presence of coagulation disorders.
4. Severe heart and lung dysfunction, including a history of angina, myocardial infarction, or other serious cardiovascular diseases.
5. Long-term use of glucocorticoids or immunosuppressants.
6. Presence or history of autoimmune diseases.
7. Presence of liver or kidney disease: alanine transaminase (ALT) and aspartate transaminase (AST) levels exceed the upper limit of normal values by more than five times or a creatinine clearance rate < 60 mL/min.
8. Allergy to IFN.
9. Presence of contraindications to IFN, including history of epilepsy or other central nervous system dysfunction, and abnormalities in the hematopoietic system (platelets <30 × 10^9^/L or neutrophils <0.5 × 10^9^/L).
10. Intake of antiviral drugs, such as acyclovir and bromouridines, within 1 week before screening period.
11. Other disqualifications as determined by researchers.

### 2.5 Drop-out criteria

During this trial, subjects experiencing the following conditions will be considered as dropout cases, and the reason and date of dropout will be recorded in a case report form.

1. Self-administration of drugs that affects the trial.
2. Occurrence of adverse events (AEs) or severe adverse events (SAEs), which makes it inadvisable to continue considering the subjects’ interests.
3. Abnormal blood routine changes (platelets <30 × 10^9^/L, neutrophil count <0.5 × 10^9^/L) during the administration of intervertebral foramen injections, leading the researchers to determine that further participation would pose a greater risk than benefit to the subjects.
4. A participant requests to withdraw consent.
5. A participant is lost to follow-up.
6. The researchers consider that further participation will be inappropriate.

### 2.6 Randomization and blinding

A statistician who does not participate in this trial will perform randomization. Random codes will be generated by SAS software using stratified block randomization. Randomization numbers will be assigned by the data analysis system for the Interactive Web Response System (DAS for IWRS).

Drug blinding will be performed by personnel from the statistical unit. A packer unrelated to the study will repackage IFN and placebo according to the drug packaging number, verification code, and the corresponding group generated by the SAS software. The investigational drug for each participant is packaged separately. The blinding process will be recorded and preserved.

All the individuals involved in the trial will be blinded to the treatment assignment until the end of the study. This study will be unblinded after the data-auditing report is completed, and the database is locked. The unblinded document will be signed jointly by the main researchers and statisticians.

Emergency letters shall be provided for emergency unblinding, and each random number corresponds to an emergency letter. If researchers consider that blinding hinders the management of AEs, emergency unblinding will be allowed.

## 3 Intervention

Participants will receive four injections into the foramen in 2 weeks according to group allocation. The injection drugs for the treatment group include 30 μg (1 mL) IFN-α1b, 50 mg lidocaine, and 2 mL physiological saline, while the control group will be treated with 30 μg placebo (1 mL), 50 mg lidocaine, and 2 mL physiological saline. The placebo is a solvent for IFN, and its packaging and appearance are consistent with those of the experimental drug. IFN-α1b injection and placebo are both produced by Sanyuan Gene Pharmaceutical Co., Ltd., Beijing, China. Every subject will be administered 150 mg of pregabalin twice daily as part of conventional therapy.

## 4 Procedure

After obtaining written informed consent, the patient will be placed in a prone position. Intervertebral foramen puncture will be performed under ultrasound guidance. After properly marking the patient and disinfecting the skin, a convex probe with a sterile cover will be placed vertically against the skin surface. Taking the injection of the thoracic intervertebral foramina as an example, the ultrasound probe is moved cephalad or caudad in a sagittal position to visualize continuous ribs. Starting from the cephalad side, the first hypoechoic area is the first rib. The probe is then moved caudally to place the target rib at the center of view. The probe is flipped 90° clockwise and slightly translated toward the thoracic vertebrae, wherein the transverse process, ribs, and pleura are visible. Subsequently, the probe is moved caudally, and the transverse process and ribs disappear, while a hyperechoic shadow formed by the inferior articular process appears. The puncture point is between the echo line of the inferior articular process and the horizontal extension of the pleura. After fixing the probe, the puncture should be performed using an in-plane approach. The needle is slowly advanced toward the target. If no blood is drawn back, the medicine is then injected.

### 4.1 Concomitant treatment

Permissible combination therapy include:

1. Oral tramadol can be administered when the VAS score of the subject decreases by <30% after receiving two intervertebral foramen injections compared to that before treatment.
2. Oral antipyretic drugs can be taken if the body temperature is >38.5°C.
3. Drugs for other diseases should be taken in the same dosage during the trial.

Disallowed concomitant treatments include:

1. Antiviral drugs, such as acyclovir and brivudine.
2. Local therapy such as lidocaine patches and capsaicin.
3. Other immune modulators such as thymosin and poly IC.
4. Taking tramadol or other opioid drugs on one’s own.
5. Antiepileptic drugs.
6. Other minimally invasive interventions, neuromodulation, acupuncture, and ozone injection.

### 4.2 Rescue medication

If the subjects are unable to withstand the pain and require analgesics, tramadol (100 mg) will be permitted as rescue medication. The patients are required to record the dosage and date of taking tramadol in a diary.

## 5 Outcome

### 5.1 Primary outcome

The VAS score and duration of pain relief will be evaluated during the entire study period as primary outcomes (Table 1). All the patients will be required to maintain a pain diary every day. The VAS is a 100 mm continuous horizontal line with the 0 mm end indicating no pain and the 100 mm end representing the most severe pain. The duration of pain relief refers to the number of days when the pain relief rate (PRR) is ≥25%. The PRR is equal to the difference between the daily VAS score after treatment and the pretreatment VAS score divided by the pretreatment VAS score.

### 5.2 Secondary outcome

#### 5.2.1 Sf-36

The SF-36 self-administered questionnaire has 36 items and will be used to evaluate health on eight multi-item dimensions, including well-being, functional status, and general health. The SF-36 scale will be completed in six visits (Table 1).

#### 5.2.2 Laboratory tests

Laboratory tests include detection and quantification of interleukin (IL)-6, IL-8, neuron-specific enolase (NSE), and S100β in the peripheral blood and VZV DNA in the peripheral blood mononuclear cells (PBMCs).

During the screening period and at the end of the fourth injection, peripheral blood samples will be collected from the subjects in disposable blood collection tubes containing ethylenediaminetetraacetic acid (EDTA) under sterile conditions. Virological test for VZV DNA in PBMCs shall be performed using polymerase chain reaction (PCR). Serological tests of IL-6, IL-8, NSE, and S100β can be done using enzyme-linked immunosorbent assay (ELISA).

#### 5.2.3 Consumption of rescue medication

The dosage, frequency, and duration of rescue drug intake will be checked and recorded at each visit.

## 6 Safety evaluation

In this trial, vital signs, electrocardiogram (ECG), hematology tests (differential count of white blood cells, neutrophils, lymphocytes, monocytes, red blood cells, and platelets), urinalysis (PH, protein, specific gravity, red blood cell microscopy, and white blood cell microscopy), and tests for blood chemical values (creatinine, urea, albumin, total bilirubin, direct bilirubin, ALT, and AST) will be conducted before and after treatment. If AEs or SAEs occur during participation, the symptoms, onset, duration, severity, relationship to the intervention, actions taken, and outcomes will be recorded in detail. Possible adverse reactions related to the treatment include fever, vomiting, nausea, granulocytopenia, thrombocytopenia, abnormal electrocardiogram, and rash. The incidence rates of AEs and SAEs will be determined.

## 7 Date management

The data administrator will design a case report form (CRF) based on the research plan and set logical verification according to the data verification plan (DVP). After passing the test and obtaining approval from the researchers, the case report will be released for further use. Data entry personnel shall input information in a timely manner into the data management system according to the CRF completion instructions. After data entry, the researcher will review and sign. After the principal researchers, the statistician and data administrator shall jointly sign the “Database Lock Record,” the data administrator will lock the database and submit it to the statistician. After the statistical analysis is completed, the data administrator will close the database.

## 8 Statistical analysis

An independent statistician will perform the full analysis set (FAS), per protocol set (PPS), and safety analysis set (SS). FAS is used to analyze data collected from patients randomized in the trial and treated at least once. PPS is performed in cases that complete all steps of the experimental protocol. SS is performed to analyze the data collected from subjects who receive at least one treatment and have a record of safety indicators. The number of cases in the SS will be determined as the denominator to calculate the incidence of AEs and SAEs.

Descriptive statistical results will be shown as mean ± standard deviation for continuous data, and as numbers and percentages for categorical data. Chi-square or Fisher’s exact tests shall be used for categorical data and independent t-tests or rank-sum tests for continuous data, depending on whether the data meet the normal distribution and homogeneity of variance. *p* < 0.05 will be considered to indicate a statistically significant difference.

For safety evaluation, the proportions of AEs and SAEs will be calculated using chi-square or Fisher’s exact tests. We will perform a paired t-test or rank-sum test to compare differences in vital signs and laboratory test results during the screening period and at the end of the intervention.

## 9 Discussion

To the best of our knowledge, our study is one of the rare and comprehensive attempts to assess the efficacy and safety of IFN-α1b injection into the foramen of patients with PHN through a randomized, double-blind, placebo-controlled multicenter clinical trial. PHN is characterized by its ease of diagnosis but difficulty in treatment. Currently, there are no drugs or methods that can completely cure PHN. Calcium channel modulators, TCAs, and other first-line drugs do not meet the needs of patients with refractory PHN. Therefore, it is necessary to develop novel treatment methods.

The etiology of PHN remains a topic of debate within the scientific community. Its development and progression are closely linked to viral replication and the subsequent immune-mediated damage to the nervous system. Following initial infection with VZV during childhood, the virus becomes latent in neurons throughout the neuraxis, including the cranial ganglia, dorsal root ganglia, and autonomic ganglia. In individuals with compromised immune systems, a reduction in VZV-specific cell-mediated immunity results in the reactivation of VZV. The acute phase of herpes is characterized by bands on the skin and pain along the spinal nerves. PHN is the most common complication of HZ after the acute phase (13–15). Investigators have proposed that VZV induces changes in sensory neurons analogous to those observed in herpes simplex infections. They further postulated that PHN may result from aberrant electrical nerve impulses triggered by viral replication within the dorsal root ganglion neurons following VZV activation (16, 17). By employing a range of techniques, researchers were able to detect VZV DNA and proteins in the PBMCs of patients with PHN. This finding revealed a notable discrepancy in the detection rate of VZV between patients with and without PHN following the healing of lesions in the acute phase of HZ. This suggests that VZV may act as a direct factor in the mechanism of PHN, which causes severe irreversible damage to the nervous system and indirectly contributes to the development of PHN (18, 19). Consequently, research into viral pathogenesis may prove pivotal in achieving long-term control of PHN.

IFN is the most important cytokine in the body’s natural antiviral immune defense mechanism initiated after viral invasion; it is an important homeostatic protein for maintaining the body’s internal environment and can act on multiple aspects of viral replication to achieve multi-targeted inhibition of viral replication (20). IFN-α1b represents a novel class of pharmaceutical agents with distinct intellectual property rights in China. These agents have a range of biological functions including antiviral, antitumor, and immunomodulatory effects. They have been endorsed as antiviral therapeutic agents in numerous consensus guidelines and clinical trials for a spectrum of diseases linked to viral infections, such as hepatitis B and condyloma acuminatum (21, 22). Its antinociceptive interactions in nociception may potentially be used in the treatment of PHN in clinical settings (23). Despite the extensive literature on the clinical efficacy of IFN-α1b in the treatment of HZ and PHN, there is a paucity of clinical studies with larger samples that could investigate the efficacy and safety of localized paraspinal administration of IFN- α1b in patients with PHN and obtain higher-quality clinical data on the use of the drug (24–26).

Compared to other treatment methods, IFN-α1b for PHN has unique mechanisms and potential benefits. First, the application of interferons targets the underlying causes of PHN, setting it apart from traditional symptomatic pain relief therapies. Second, while interferons can have side effects, their risk of addiction and associated adverse effects is generally lower compared to stronger analgesics, such as opioids. Furthermore, although this study focuses exclusively on PHN patients, previous data has shown that interferons can be effectively administered at various stages following a herpes zoster outbreak, thereby providing a flexible approach to meet the treatment needs of diverse patients (24, 26).

As PHN is a chronic pain disorder, long-term pain management and control is necessary. A single visit by the patients to the clinic serves to control current pain, which is the primary complaint. Consequently, the efficacy endpoints of this study are designed to focus on VAS scores. Furthermore, continuous improvement in pain scores during the long-term follow-up shall be used to assess the duration of continuous pain control. Any pain experienced during the follow-up period will be recorded along with its intensity. A reduction of ≥25% in the VAS score and treatment effectiveness of ≥40% are considered clinically significant. In accordance with ethical considerations, this study discourages alterations to patient medication regimens. Instead, it will document changes in the dosage of medication taken by subjects in relation to pain associated with PHN in the short-term and during follow-up. If this study yields positive results, it may be possible to recommend the implementation of intervertebral foraminal injection techniques in the treatment regimen of patients with PHN and justify the undertaking of new studies on cost-effectiveness.
